# Patients Prefer Being Offered a Mirror to See Their Cervix and External Genitalia During Well-Exams while Clinician Perceptions May Create Barriers to Offering a Mirror: A Mixed Methods Study in a Primary Care Setting

**DOI:** 10.1089/whr.2025.0030

**Published:** 2025-05-12

**Authors:** Luci Olewinski, Stephanie Hartmann, Savannah McKenzie, Shannon Lewis, Grace Saxon, Robert Eric Heidel, Fatima Ahmed

**Affiliations:** ^1^ University of Tennessee Graduate School of Medicine, Department of Family Medicine, Knoxville, Tennessee, USA.; ^2^ Piedmont Healthcare Inc., Department of Family Medicine, Newnan, Georgia, USA.; ^3^ University of Tennessee Medical Center, Knoxville, Tennessee, USA.; ^4^ University of Tennessee Graduate School of Medicine, Department of Surgery, Knoxville, Tennessee, USA.

**Keywords:** mirror pelvic exam, patient-centered care, patient preferences, well exam

## Abstract

**Objective:**

Patient-centered care is a core value of both Family Medicine and Obstetrics & Gynecology. We sought to know if patients prefer being offered a mirror to see their cervix and external genitalia during asymptomatic speculum exams (Mirror Pelvic Exam, MPE). Additionally, we explored clinicians' (medical assistants, residents, and faculty) opinions about offering patients a mirror during exams.

**Methods:**

The patient portion was a cross-sectional mixed-methods survey of people presenting for cervical cancer screening at a residency-based Family Medicine Clinic. Patients took a presurvey, were offered a mirror to see their external genitalia and/or their cervix during the exam, and then took a post-survey. The clinician portion was a mixed-methods survey given at the initiation of the study and after the four-month patient survey period.

**Results:**

While only half the patients (*n* = 22) accepted the use of a mirror, the majority preferred being offered and felt offering a mirror should be a routine part of the well-exam. Being offered a mirror did not bother anyone. Free responses further emphasized that patients preferred being offered the MPE. Over half of clinicians (*n* = 51) felt the MPE was a good idea, but only a third felt it would improve patient satisfaction. Some did not offer the MPE due to thinking patients would not want the option, lack of comfort with the MPE, or concerns about slowing down clinic flow.

**Conclusion:**

Patients prefer being offered the MPE while clinicians did not have insight into patients’ preferences. Our results suggest clinician perceptions about offering the MPE are a barrier to the MPE as a standard of care.

## Introduction

The dual purposes of this study are to discover whether patients prefer being offered a mirror to see their cervix and external genitalia during asymptomatic speculum exams and clinician perceptions of the mirror pelvic exam (MPE). Anecdotes in historical texts document an increase in the popularity of teaching women to use mirrors and plastic speculums to self-examine as part of the feminist movement.^1–3^ Some medical and nursing texts also recommend offering a mirror as part of a pelvic exam (MPE).^4–6^ The MPE is suggested to help with anxiety, knowledge, and empowerment;^3,5,6^ however if patients prefer being offered a mirror and if offering and acceptance affect outcomes have been sparsely studied. A 1979 survey published in the Journal of Family Practice found the majority of the 977 women surveyed wanted to know more about the pelvic exam and their own anatomy, but only 46.5% would want to use a mirror if it was offered.^7^ In a study where women were randomized to receive a standard pelvic exam versus an MPE with a focus on education about anatomy and the exam, 87.7% said using the mirror made them feel more relaxed and 80.6% would choose this option in the future.^8^ A 1989 German study (with an abstract available in English) reported greater satisfaction with the well woman exam when a mirror is used, but also found a link between patients accepting a mirror and their own pre-exam comfort with their bodies.^9^ In a more contemporary study of women presenting to a gynecological clinic for vulvar pathology, women were allowed to choose to use a mirror to see their external genitalia, and those who accepted said they would prefer this option in the future. Acceptance resulted in enhanced levels of control and knowledge but not pain or vulnerability.^10^

Despite prior studies indicating the benefits of incorporating the MPE, it has not become standard in clinical practice. Critics of the use of MPE propose concerns about the difficulty of the utilization of the mirror during the exam, the expense of stocking mirrors in the clinic, hygiene concerns, and prolonged exam time.^10^ Data is also lacking regarding the attitudes of physicians, nurses, and medical assistants regarding the MPE. Knowing and incorporating patient preferences is in line with patient-centered care, a core value of both Family Medicine and Obstetrics & Gynecology,^11^ so our main outcome measure of the patient survey was a patient preference in being offered a mirror to see their external genitalia and cervix during a well exam. Secondary outcomes were acceptance rates and clinician perceptions of offering the MPE.

## Materials and Methods

### Study design

Our study is a novel study based on a review of the survey procedures used by Tam et al,^10^ inclusion of the Female Genital Self-Image Scale (FGSIS),^12,13^ and discussion with our nursing staff and physicians. The patient portion was a cross-sectional survey of people with cervixes and female genitalia presenting for well exams (asymptomatic patients having cervical cancer screening with a speculum exam). We utilized pre- and post-exam surveys to assess patient characteristics and acceptability of the intervention. The clinician portion was pre-and post-intervention assessments of physician and medical assistant attitudes towards the MPE (Supplementary Data S2).

### Survey tool

The patient-focused survey had two parts (Supplementary Data S1). The first was a pre-exam survey incorporating the 4-question FGSIS-4 and prior exposure to the use of a mirror during a well woman exam. The FGSIS-4 is a validated scale that positively correlates female genital self-image with sexual function.^13^ We included the FGSIS-4 to see if there was any connection between comfort with one’s anatomy and acceptance/preference to be offered a mirror.^9^ Patients were offered a mirror to see their external genitalia prior to the speculum exam and again after the speculum was in place to see their cervix. They then took a post-experience survey to assess acceptance or refusal, and if they prefer being offered a mirror during the exam. We aimed to design an efficient survey to answer the question of whether patients prefer being offered a mirror, and so we considered but then ultimately excluded questions about pain, vulnerability, a sense of control, and comfort to avoid a survey that would fatigue the patients. There was a free-response section for patients to explain their responses. As the goal of our initial survey is to know if people with cervixes, as a universal group, should be offered a mirror during an exam, we did not include questions about age, race/ethnicity, prior pathology, or pregnancy. A potential future study could identify which demographic groups prefer using a mirror during the exam, but this was not our current study’s purpose.

There were also longitudinal surveys of nursing staff, faculty, and resident physicians (clinicians) before the start of the patient survey administration period (after education about the process) and then after the patient survey collection period was completed (Supplementary Data S2). The clinician-focused surveys asked about opinions on the burdens and benefits of offering mirrors. These were given prior to the study roll-out in January and after study completion in June.

After survey creation, they were reviewed by the Graduate School of Medicine statistician, our research coordinator, and a librarian with expertise in plain language use in surveys. The patient survey was then tested with five clinic patients and the clinician survey was tested with two medical assistants not working in our clinic and two OB/gyn’s in our system’s residency program with no changes recommended. The patient survey was then translated into Spanish by a professor of Spanish language with prior experience translating medical documents for our clinic.

This study was approved by the Institutional Review Board (IRB) at the University of Tennessee Graduate School of Medicine, approval number 5001.

### Participants

The survey was offered to any person fluent in English or Spanish presenting to our Family Medicine residency clinic for asymptomatic cervical cancer screening over the age of 18 between February 1 and May 31, 2023. Not every patient who has cervical cancer screening at our clinic identifies as a woman, so we prefer to describe our participants as patients. Exclusion criteria were non-English or Spanish-speaking patients, any inability to complete the survey, or any physical or cognitive condition that would affect the participant’s ability to visualize the mirror during the examination.

Exclusion criteria for clinicians included anyone who did not participate in pap smears or well exams.

### Data analysis

We hypothesized that less than 10% of patients would have used a mirror to see their cervix previously, over half of patients offered a mirror will accept the mirror, over half of patients will prefer being offered a mirror, and clinicians will be hesitant at first but more comfortable after they have incorporated offering a mirror into the exam.

Frequency and percentage statistics were used to describe the responses to survey questions. Chi-square and Fisher’s Exact Test were used to compare independent groups on survey responses. Statistical significance was assumed at an alpha value of 0.05 and all analyses were performed using SPSS Version 29 (Armonk, NY: IBM Corp.).

Open-ended responses were sorted into descriptive themes by one member of the study team. Two other team members then checked the themes against the specific responses to ensure agreement, and then each response was assigned an appropriate theme(s). A fourth research team member then assessed the themes and agreed with the classifications.

## Results

Twenty-two patients participated in the study, average age of 40 (SD 13.9). This was estimated to be potentially a third of patients who had well exams during the four-month study period of Feb 1 to May 31 based on billing for 221 pap smears during the same academic year. Eleven patients accepted the MPE, with one of the 11 only using the MPE to see their cervix but not their external genitalia. Eleven patients did not accept the MPE. One patient checked the box indicating that they had accepted the mirror, but as all their other responses indicated they had not used the mirror, this patient was coded as having declined the MPE. In the pre-survey, eight patients had used a mirror to see their external genitalia before (36.4%), and two had seen their cervix before (9.1%). Of patients who had seen their external genitalia before, five accepted the MPE, and three declined.

In the patient post-survey, all who accepted could see their external genitalia and cervix. Of all 22 patients, 17 (77.3%) said they liked being offered a mirror (95% of the 18 respondents to that question; four patients had left that question blank). Twenty patients (90.9%) said being offered a mirror did not bother them (100% of 20 respondents to that question) and 19 (86.3%) said being offered a mirror should be a routine part of the exam (95% of 20 respondents to that question). The two patients who did not respond and the one patient who disagreed that being offered a mirror should be a routine part of the exam all declined to use the mirror.

Our patients had mainly positive responses to the FGSIS-4 scores (Table 1) so we were not able to assess the impact of scores on opinions about the MPE. The three people who disagreed with any of the FGSIS-4 questions all declined the MPE, but this was not statistically significant (*p* = 0.20).

**Table 1.** Patient Female Genital Self-Image Scale Scores

	Did not respond	Strongly disagree	Disagree	Agree	Strongly agree
I am satisfied with the appearance of my genitals	18%	0	5%	59%	18%
I would feel comfortable letting a sexual partner look at my genitals	9%	0	5%	68%	18%
I think my genitals smell fine	9%	0	0	73%	15%
I am not embarrassed about my genitals	9%	0	14%	55%	23%

Fifty-two percent of residents (13 of 25), 25% of faculty (3 of 12), and 29% of medical assistants (4 of 14) were able to help patients see their external genitalia; 40% of residents, 25% of faculty, and 29% of medical assistants were able to help patients see their cervixes. Aside from a reduction in concerns about cleanliness, opinions about the MPE did not change significantly after the study period (Table 2).

**Table 2.** Clinician Pre- and Post-Responses

Agree with:	Pre	Post	*p* value	Comments
Received enough training	68%	82.7%	0.09	
Concerns about cleanliness	30%	5.8%	0.002	
Concerns about dropping	22%	11.5%	0.06	
MPE good idea (ext gen)	68%	75%	0.49	Results skewed by four ambivalent write ins.
MPE good idea (cervix)	74%	73.1%	0.49	Results skewed by two ambivalent write ins.
MPE good use of time	60%	51.9%	0.26	
MPE increase patient satisfaction	34%	36.5%	0.14	
MPE improve health outcomes	30%	28.8%	0.18	

MPE, Mirror Pelvic Exam.

While clinicians were not as likely to agree the MPE would improve patient satisfaction as they were to think it was a good use of time or a good idea, only agreeing the MPE would improve patient satisfaction had an association with successfully helping patients to see their external genitalia (p:eg < 0.03) and cervix (p:*c* < 0.003) during the MPE. Clinician role was not associated, nor was agreeing on the post-survey that they had enough training, concerns about the cleanliness of the mirror, concerns about dropping the mirror, that the MPE to see external genitalia or cervixes was a good idea, that the MPE was a good use of time, or that the MPE would improve patient outcomes.

### Open-ended results

Both surveys had open-ended questions to better understand people’s perspectives on the MPE. Eight of the ten patients (Table 3) who declined the MPE wrote in reasons of disinterest, time, comfort, or concerns that they would be unable to participate due to habitus, positioning, or the size of the mirror. Patients wrote they accepted out of curiosity or wanting knowledge about their own bodies, or to be helpful to the study. Half of the acceptors had some difficulty seeing that was overcome by positioning, angling of the mirror, or explanation of anatomy by their doctor. All patients who wrote in liked being offered the mirror; those that declined did not accept due to disinterest or lack of concern, with one respondent wishing there was a bigger mirror. We used a hand mirror, but other studies have used larger birthing mirrors for the MPE.^10^ Empowerment, body positivity, and interest (with subgroups of curiosity and empowerment) were given by patients that accepted as reasons why they liked being offered a mirror, why being offered did not bother them, and why they thought being offered a mirror should be a routine part of the pap exam.

**Table 3.** Patient Free-Response Sections

**Why did you accept or decline the mirror?**
*Non-acceptors*:
I didn’t feel comfortable seeing what was going on and I didn’t want to delay the process
I don’t want to pass out
I have no interest in seeing what she looks like
No reason really just have no interest in looking at it
No need to see
No interest
Maybe when I was younger
Don’t know if I could see in small handheld mirror
* Acceptors:*
To see what everything looked like
I would like to see what my cervix looks like, I’ve never seen it
Wanted to see my cervix
I accept
Thought it would be cool to see down there
I wanted to see my cervix
I wanted the chance for more information about my body
In the interest of science and curiosity
Because I want to know how I work on the inside
To help with study
**If you did use the mirror, please let us know what helped or stopped you from seeing.**
The nurse angling the mirror as I requested
Better when you can hold the mirror yourself
The mirror helped in seeing everything but speculum was kind of in the way
The paper was in the way but once I moved it I could see clearly
The mirror and doctor assistance pointing it out
Someone holding me up
**Please use this space to explain your response to if you liked being offered the mirror:**
* Non-acceptors:*
Just being given the option was nice
If someone is nervous about the process it might help them feel better
If I was concerned, this would definitely be an option
Just not interested in that part of my body
Well if I wanted to watch I would need big mirror
* Acceptors:*
I think it’s a great option to be able to know
It made me feel like the doctors celebrated and normalized experiencing our genitalia
Because you never see where your babies come from. It’s amazing when you see where
I learned a little more about my body
No harm to see my own anatomy
Never seen my cervix before was interested in what it looked like
I’ve never really seen my genitals from that angle
It was nice to see a part of my body I’ve never seen
It was cool to see myself
It was interesting to see my cervix
**No one felt being offered a mirror bothered them, only acceptors responded to why it did not bother them:**
It was nice to see my body in a way that I never had before
Thought it was less bothersome and more educational
It’s amazing to see how baby’s are born (happy face drawing)
Because I want to understand my body more
[Doctor] explained everything before he did it
The attitude of [doctor] and [nurse]. Matter of fact. Positive
**Do you think being offered a mirror should be a routine part of the well woman exam?**
* Non-acceptors:*
Give choice
For many people they don’t know what’s going on and don’t know about their genitalia and it can be a great learning opportunity
* Acceptors:*
Being offered the mirror allows for the option, and one does not have to accept is they do not want to
It’s up to the female
The offer is positive and respectful
To normalize it
Makes paps more comfortable
More autonomy and see what they are looking at
So I can know what he was looking for and know the right way to have it done
My mother couldn’t explain it to me and my doctor can and show me
I think women should have the opportunity to be familiar with their parts
**What else would you like to tell us?**
* Non-acceptors:*
I think the mirror is a great idea for some. I have done this many times and just want to get it over with big lighted mirror with magnification for over 50+ women TY
* Acceptors:*
Great study. Thanks!
Thank you
Was very thrilled to see what was happening down there during the procedure
It was a great experience
None!
Great experience, and I hope more women learn and understand their bodies. It’s a great thing to do and just see where life started for your kids

Very few open-ended responses were given for the clinician pre-survey, all by medical assistants. Two wrote that they did not think patients would be interested due to wanting the exam to be over quickly, and one had concerns about whether the physician versus the medical assistant would be in charge of offering the mirror. The clinician post-survey responses to questions about whether their perceptions had changed or if there was anything else they wanted to say fell into themes of interest (either the patients were not interested in the MPE, or the clinicians themselves forgot to offer; as well as surprise that patients were so interested in the study and their anatomy) opportunity (they did not have a pap during the study period); learning (that patients enjoyed learning or that clinicians thought patients learning about their anatomy was important); comfort (some clinicians felt uncomfortable with offering the mirror), time (some did not offer the MPE because they did not want to delay clinic flow); and responsibility (medical assistants felt it was the doctor’s role to offer the survey, doctors felt it should be the medical assistant’s role, and so neither offered). Of 27 total write-in responses to these two questions, 10 commented on patients not wanting to use the mirror and eight expressed that they themselves did not offer the MPE-either out of lack of interest in the study or feeling uncomfortable with offering patients the mirror.

## Discussion

The major findings of this study are that patients preferred being offered an MPE while physicians and medical assistants did not have insight into this preference. Studies done periodically over the past five decades have shown that patients prefer being offered a mirror and that there are outcome benefits to the MPE. Our study adds to the body of research on the MPE by assessing clinician opinions in addition to patient preferences. Failure of the MPE to become standard clinical practice may be based on the clinician’s viewpoint rather than patient preference.

Our clinician post-survey free responses tended towards the negative. We estimate that only a third of potential patients were surveyed during our study period; this may have been due to some clinicians not offering the survey to their patients. However, some clinician free responses commented on being surprised at how much patients liked being offered a mirror. It cannot be known if the universal offering of the survey and MPE would have resulted in more patients saying they did not want to be offered a mirror or if the trend toward positive reception of the MPE would have continued. Clinician reluctance was an unexpected variable in the administration of this research project, but with the unpredictability of anticipating each potential well speculum exam in our Family Medicine clinic across more than 50 clinicians (medical assistants, residents, and faculty) we were not able to assure universal offering of the survey and MPE.

While only half of the surveyed patients accepted the MPE, most preferred being offered the mirror. A patient-centered approach to clinical care is a core value of Family Medicine,^11^ and our results offer even more support for offering the MPE. Part of our motivation to do this study was to know why the MPE is not universally taught nor offered in practice despite previous research supporting patients’ preference for being offered this exam.^8,10,14^ Clinician reluctance may help explain why this is not routinely taught and not yet routinely offered in the clinical setting, despite recommendations to offer the MPE in resources used for teaching the pelvic exam.^4–6^

The Female Genital Self-Image Scale has been shown to be predictive of gynecological exam behavior, in that surveyed women with higher FGSIS scores were more likely to have received a gynecological exam in the last 48 months.^12^ We chose to include this scale in our survey to assess whether genital self-image may affect a patient’s desire to view their own anatomy and/or to be offered a mirror. Since the FGSIS-4 scores of patients in our study were nearly universally positive, we could not explore the association between the FGSIS and preferences about the MPE.

### Limitations and during-study troubleshooting

During the study period, we had monthly meetings to assess the project. After the first month of the project, we found there was confusion as to whether the point of the study was the MPE itself or offering the survey. Clinicians had not been offering the survey to patients they felt were not likely to elect for an MPE. Once we discussed this in nursing, resident, and faculty meetings clinicians began to offer the survey more consistently. Clinicians also noted that some patients had difficulty with the MPE due to body size or core strength but that raising the head of the exam table or working on mirror angling helped. We feel this experience is useful as it replicates what might happen if a clinic were to set a goal of incorporating the MPE and uncovers some potential real-world barriers to the MPE as standard practice.

After using the novel survey tool, we noted that it did not capture some useful information. We could not tell if the inability to help patients see their external genitalia and cervix was because clinicians did not offer the MPE or because they offered and were not successful during the MPE. However, for our population, all patients who accepted did say they successfully saw. We also did not ask clinicians on the post-survey if they would change their practice to offering the MPE. Our questions on thinking this was a good idea, a good use of time, or likely to improve patient satisfaction can hint at clinician preference, but asking directly would be more helpful to know if clinicians would change their practice to match patient preference.

## Conclusions

Patients prefer being offered the MPE. Clinicians (nursing, residents, faculty) tended to agree the MPE was a good idea but did not have insight into patients’ preferences to be offered the MPE. Our results suggest clinician beliefs are a barrier to the implementation of more widespread use of MPE.

## Abbreviations Used

FGSIS: Female Genital Self-Image Scale
MPE: Mirror Pelvic Exam

## References

[1] Bittiker A. Making visible the invisible: A brief history of gynecological practice and representation. University of Florida Journal of Undergraduate Research 2002;4(4).

[2] Boston Womens Health Collective. Our Bodies Ourselves. Boston: New England Free Press; 1971.

[3] Murphy M. Immodest witnessing: The epistemology of vaginal self-examination in the US feminist self-help movement. Feminist Studies 2004;30(1):115–147.

[4] Bates CK, Carroll N, Potter J. The challenging pelvic examination. J Gen Intern Med 2011;26(6):651–657. 21225474 10.1007/s11606-010-1610-8 PMC3101979

[5] Williams AA, Williams M. A guide to performing pelvic speculum exams: A patient-centered approach to reducing iatrogenic effects. Teach Learn Med 2013;25(4):383–391. 24112210 10.1080/10401334.2013.827969

[6] Simms-Cendan J. Examination of the pediatric adolescent patient. Best Pract Res Clin Obstet Gynaecol 2018;48:3–13. 29056510 10.1016/j.bpobgyn.2017.08.005

[7] Petravage JB, Reynolds LJ, Gardner HJ, et al. Attitudes of women toward the gynecologic examination. J Fam Pract 1979;9(6):1039–1045. 521765

[8] Liston J, Liston EH. JR,. The mirror pelvic examination: Assessment in a clinic setting. JOGN Nurs 1978;7(2):47–49. 10.1111/j.1552-6909.1978.tb00907.x 246478

[9] Blaser A, Werren S, Brügger D, et al. The mirror as a possibility of participation in routine gynecologic examination: Effects on the psychological status of the female. Gynakol Rundsch 1989;29(2):69–77. 2737538

[10] Tam T, Crisp CC, Hill AM, et al. Utilization of a mirror during pelvic examinations: Does it improve the patient’s experience? Female Pelvic Med Reconstr Surg 2021;27(3):208–213. 33620906 10.1097/SPV.0000000000000975

[11] Hudon C, Fortin M, Haggerty JL, et al. Measuring patients’ perceptions of patient-centered care: A systematic review of tools for family medicine. Ann Fam Med 2011;9(2):155–164. 21403143 10.1370/afm.1226 PMC3056864

[12] DeMaria AL, Hollub AV, Herbenick D. The female genital self-image scale (FGSIS): Validation among a sample of female college students. J Sex Med 2012;9(3):708–718. 22240088 10.1111/j.1743-6109.2011.02620.x

[13] Herbenick D, Schick V, Reece M, et al. The female genital self‐image scale (FGSIS): Results from a nationally representative probability sample of women in the United States. J Sex Med 2011;8(1):158–166. 21044269 10.1111/j.1743-6109.2010.02071.x

[14] Willard MD, Heaberg GL, Pack JB. The educational pelvic examination women’s responses to a new approach. J Obstet Gynecol Neonatal Nurs 1986;15(2):135–140. 10.1111/j.1552-6909.1986.tb01378.x 3634793

