# Supplementary material for: Patients Prefer Being Offered a Mirror to See Their Cervix and External Genitalia During Well-Exams while Clinician Perceptions May Create Barriers to Offering a Mirror: A Mixed Methods Study in a Primary Care Setting
Source: Womens Health Rep (New Rochelle). 2025 May 12;6(1):556–63. doi: 10.1089/whr.2025.0030 (PMC12177316; doi:10.1089/whr.2025.0030)
Supplement: Supplementary Data S1 [file whr.2025.0030_supplementary_data_s1.docx]

The University of Tennessee Knoxville Family Medicine Residency and University Family Physicians are asking you to take a research survey about your thoughts on being offered a mirror during a well exam. We will use this information to help guide how we do our exams here. We hope to publish this data to inform other clinics about what patients prefer.

Taking part is voluntary. You can fill out the research survey even if you choose not to use a mirror during the exam. Filling out part of the research survey does not mean you have to complete the whole survey.

Your doctor may ask you during the exam if you would like to use a mirror to see your external genitalia and/or cervix. You can say yes or no. Filling out this survey does not mean you have to use the mirror.

If you have taken the survey before, please tell us. You may request a mirror without completing the survey again.

If you consent to participate in this research survey, please fill out the first page before your exam, and the second page after your exam.

**Pre-Exam Survey**

1. Age:  ________

2. Is this your first pelvic exam?  🞏 Yes    🞏 No

3. Have you ever used a mirror to see your external genitalia before? 🞏 Yes    🞏  No     🞏 Unsure

4. Have you ever used a mirror to see your cervix before?  🞏 Yes      🞏 No     🞏 Unsure

5a. I am satisfied with the appearance of my genitals.

🞏 Strongly Disagree 🞏 Disagree 🞏 Agree 🞏 Strongly Agree

5b. I would feel comfortable letting a sexual partner look at my genitals.

🞏 Strongly Disagree 🞏 Disagree 🞏 Agree 🞏 Strongly Agree

5c. I think my genitals smell fine.

🞏 Strongly Disagree 🞏 Disagree 🞏 Agree 🞏 Strongly Agree

5d. I am not embarrassed about my genitals.

🞏 Strongly Disagree 🞏 Disagree 🞏 Agree 🞏 Strongly Agree

**Post-Exam Survey**

Now that you have been offered a mirror:

6. Did you accept?

🞏 Yes 🞏  No 🞏 Yes, but For External Genitalia Only 🞏 Yes, but For Cervix Only

7.Why did you accept or decline the mirror? ____________________________________________

________________________________________________________________________________

If you did use the mirror: 

8a. Were you able to see your external genitalia? 🞏 Yes    🞏 No     🞏 N/A    🞏 Unsure

8b. Were you able to see your cervix?   🞏 Yes    🞏 No     🞏 N/A    🞏 Unsure

8c. Please let us know what helped or stopped you from seeing.  ____________________________

________________________________________________________________________________

Please answer the following questions even if you did not use the mirror.

9a. Did you like being offered a mirror to see your cervix?  🞏 Yes     🞏 No

9b. If you would like, please use this space to explain your response. ________________________

 _______________________________________________________________________________

10a. Did this experience bother you in any way?  🞏 Yes     🞏 No

10b.If you would like, please use this space to explain your response.  _______________________

_______________________________________________________________________________

11a. Do you think being offered a mirror should be a routine part of the well woman exam?

🞏 Yes     🞏 No

11b. If you would like, please use this space to explain your response. _______________________

_______________________________________________________________________________

12. What else would you like to tell us? Please use the back of this page if you need more space.

________________________________________________________________________________

________________________________________________________________________________

________________________________________________________________________________

________________________________________________________________________________
