# Supplementary material for: Patients Prefer Being Offered a Mirror to See Their Cervix and External Genitalia During Well-Exams while Clinician Perceptions May Create Barriers to Offering a Mirror: A Mixed Methods Study in a Primary Care Setting
Source: Womens Health Rep (New Rochelle). 2025 May 12;6(1):556–63. doi: 10.1089/whr.2025.0030 (PMC12177316; doi:10.1089/whr.2025.0030)
Supplement: Supplementary Data S2 [file whr.2025.0030_supplementary_data_s2.docx]

The University of Tennessee Knoxville Family Medicine Residency and University Family Physicians are asking you to take a research survey about your thoughts on offering patients a mirror during a well woman exam. Taking part in this survey is voluntary. Filling out part of the survey does not mean you have to complete the whole survey. We will use this information to help guide how we do our exams here. We hope to publish this data to inform other clinics about what patients prefer.

**Well Exam Mirror Study: Provider Pre- Survey**

1. Age _____________

2. Do you have or have you ever had a cervix?  🞏 Yes  🞏 No

3. Role at UFP: _______________

4. Do you feel like you received enough training to help women use the mirror?  🞏 Yes    🞏 No

5. Do you feel comfortable offering a patient a mirror during a genital exam? 🞏 Yes     🞏 No

6. Do you have concerns about the cleanliness of the mirror? 🞏 Yes     🞏 No

7. Do you have concerns about dropping the mirror? 🞏 Yes     🞏 No

8. Do you think it’s a good idea to offer women mirrors to see their external genitalia?

🞏 Yes  🞏 No

9. Do you think it’s a good idea to offer women mirrors to see their cervix?  🞏 Yes     🞏 No

10. Do you think offering a mirror is a good use of time in the visit?  🞏 Yes     🞏 No

11. Do you think offering a mirror will increase patient satisfaction?   🞏 Yes     🞏 No

12. Do you think offering a mirror will improve health outcomes for patients?   🞏 Yes     🞏 No

13. Anything else we should know? ________________________________________________

_____________________________________________________________________________

_____________________________________________________________________________

_____________________________________________________________________________

**Provider Post-Survey**

1. Age ____________

2. Do you have or have you ever had a cervix?  🞏 Yes     🞏 No

3. Role at UFP: _____________

4. Do you feel like you received enough training to help women use the mirror?   🞏 Yes     🞏 No

5. Were you able to help women use the mirror to successfully see their external genitalia?

🞏 Yes     🞏 No

6. Were you able to help women use the mirror to successfully see their cervix?  🞏 Yes     🞏 No

7. Did you have concerns about the cleanliness of the mirror?  🞏 Yes     🞏 No

8. Did you have concerns about dropping the mirror?  🞏 Yes     🞏 No

9. Do you think it’s a good idea to offer women mirrors to see their external genitalia? 🞏 Yes     🞏 No

10. Do you think it’s a good idea to offer women mirrors to see their cervix? 🞏 Yes     🞏 No

11. Do you think this was a good use of time in the visit?  🞏 Yes     🞏 No

12. Do you think this increased patient satisfaction?  🞏 Yes     🞏 No

13. Do you think this improved health outcomes for patients?  🞏 Yes     🞏 No

14. If your perceptions about offering a mirror during a well exam have changed since the initial survey, please tell us how and why:

_____________________________________________________________________________

_____________________________________________________________________________

_____________________________________________________________________________

15. Anything else we should know?

_____________________________________________________________________________

_____________________________________________________________________________

_____________________________________________________________________________

_____________________________________________________________________________

_____________________________________________________________________________
